# The effects of dutasteride and tamsulosin, alone or in association, in the kidney morphology: experimental study in rats

**DOI:** 10.1590/acb410826

**Published:** 2026-03-09

**Authors:** Marcello Henrique Araujo da Silva, Simone Gomes Ferreira, Bianca Martins Gregório, Francisco José Sampaio, Diogo Benchimol de Souza

**Affiliations:** 1 Universidade do Estado do Rio de Janeiro – Urogenital Research Unit – Rio de Janeiro (RJ) – Brazil.

**Keywords:** Prostatic Hyperplasia, Dutasteride, Tamsulosin, Kidney

## Abstract

**Purpose:**

To evaluate the renal morphology after isolated and combined administration of dutasteride and tamsulosin in rats.

**Methods:**

Forty male rats were divided in groups: control group; D, receiving 0.5 mg/kg/day of dutasteride; T, receiving 0.4 mg/kg/day of tamsulosin; and D+T, receiving both dutasteride and tamsulosin. The drugs were given by gavage for 40 days. The animals were killed, and kidneys were collected for stereological analyses of kidney volume, cortical volume, volumetric density of glomeruli, mean glomerular volume, and number of glomeruli per kidney. Data was compared by analysis of variance followed by Bonferroni’s post-hoc test.

**Results:**

Kidney weight, kidney volume, cortical volume, and glomerular volumetric density were reduced in all treated groups, in comparison to control group. The mean glomerular volume was reduced in groups D and D+T, in comparison to control rats, but in group T this parameter was not altered. Finally, the number of glomeruli per kidney was reduced by 37.9% in the group D, by 25.7% in group T, and by 33.07% in group D+T, in comparison to control group.

**Conclusion:**

The use of dutasteride and tamsulosin (isolated or in combination) promoted kidney damage with nephron losses in the rodent model. Animals that received dutasteride showed more severe renal modifications than those that received tamsulosin and combined therapy.

## Introduction

Benign prostatic hyperplasia (BPH) results in a prostatic enlargement with urinary symptoms which negatively impacts quality of life1. This condition affected 50% of men older than 50 years old and 90% of men in their 80s^1,2^. One of the most used treatments for BPH is the prescription of 5-alpha reductase inhibitors (5-ARIs)^3^. The inhibition of testosterone to be converted to dihydrotestosterone (DHT) normally reduces the prostate volume, ameliorating the voiding symptoms^2^.

Some side effects are associated with the use of 5-ARIs. The most mentioned adverse effects involve sexual function^4^. The reduction of libido and erectile dysfunction associated with 5-ARIs are well documented, and histomorphometrical alterations of the corpus cavernosum have been previously described^4–8^. However, recently it has been reported that finasteride and dutasteride promote renal morphological and functional damages in the rodent model^9–11^.

Other medical option for BPH treatment is the use of tamsulosin, an alpha-1 adrenergic antagonist, which can be used alone or in association with dutasteride^12^. Tamsulosin acts by relaxing smooth muscle, and ameliorating the urinary symptoms associated with BPH^13^. Even so, the use of this drug can lead to adverse effects including ejaculation dysfunction^14^. The impact of tamsulosin, alone or in association with dutasteride, on kidney morphology has not been investigated yet.

The hypothesis of this study was that the concomitant use of dutasteride and tamsulosin may result in kidney morphological renal alterations, as well as in the isolated use of dutasteride. Thus, the aim of this study was to evaluate, in a rodent model, the renal morphology after isolated and combined administration of dutasteride and tamsulosin.

## Methods

Forty male Wistar rats were used in this study. All animals were created in our laboratory and were included in the experiment with 4 months of age. They were kept in a room with a controlled temperature (mean ± standard deviation 25°C ± 1°C) and artificial dark-light cycles (lights on from 7 a.m. to 7 p.m.) and had free access to standard rat food and water.

This project was formally approved by the local ethics committee (Instituto de Biologia Roberto Alcântara Gomes Committee for Ethical Use of Animals), under the protocol number CEUA-057/2018. All experiments were conducted at the Urogenital Research Unit, Department of Anatomy, Universidade do Estado do Rio de Janeiro.

The animals were randomly divided into the following groups:

- Control group (n = 10), which received distilled water;
- Group D (n = 10), which received 0.5 mg/kg/day of dutasteride (Avodart, GlaxoSmithKline Pharmaceuticals, Poznan, Polony)5,6,10;
- Group T (n = 10), which received 0.4 mg/kg/day of tamsulosin (Secotéx, Astellas Pharma, Meppel, Netherlands)15,16;
- Group D+T (n = 10), which received the combination of 0.5 mg/kg/day of dutasteride and 0.4 mg/kg/day of tamsulosin (Combodart, GlaxoSmithKline Pharmaceuticals).

All administrations were given by gavage (diluted to the same final volume), during 40 consecutive days.

After 40 days of experiment, all animals were killed by an isoflurane overdose. The kidneys were dissected and weighed, and their volumes were measured by Scherle’s method^10,17,18^ immediately before fixation by immersion in 4% buffered formaldehyde for a minimum period of 24 hours.

Left kidneys were transversely sectioned sequentially, with a thickness of 2 mm (for each section). These sections were used for determining the cortical-to-medullary ratio using Cavalieri’s principle^19,20^. The absolute cortical volume (CV) was calculated by multiplying the cortical-medullary ratio by the renal volume.

Fragments from the right kidneys cortical area were collected using the orientator method^21^ and routinely processed for paraffin embedding. Sections of 5-µm thickness were obtained and stained by hematoxylin and eosin. Twenty-five randomly selected histological fields of the cortex of each rat were analyzed. These fields were photographed using a digital camera (DP70, Olympus, Tokyo, Japan) coupled with a microscope (BX51, Olympus, Tokyo, Japan) under 200× magnification^10,18,20,22^.

Glomerular volumetric density (Vv[glom]), which indicates the proportional volume occupied by the glomeruli in the cortex, was estimated by the point counting technique using a M42 test system. Volume-weighted glomerular volume (VWGV), which indicates the mean volume of the glomeruli, was estimated by the point-sampled intercepts method by analyzing 50 glomeruli per animal. Quantitative analyses of Vv[glom] and VWGV were performed using the ImageJ software (version 1.46r, National Institutes of Health, Bethesda, MD, United States of America). The total number of glomeruli per kidney was estimated by dividing the product of the CV and Vv[glom] by VWGV^10,18,20,22^.

One-way analysis of variance (ANOVA) with Bonferroni’s post-hoc test was used to compare mean values, with the significance level set at *p* < 0.05. All analyses were performed using the GraphPad Prism software (version 5.0, San Diego, CA, United States of America).

## Results

Kidney weight in the groups D, T and D+T was reduced by 14.6, 10.8, and 8.5% in comparison to control group. Kidney volume was also reduced, by 15.4% in group D, by 10.6% in group T, and by 8.9% in group D+T, in comparison to control group. Among the groups D, T, and D+T, no difference was observed.

Cortical-to-medullary ratio was decreased by 11.3, 9.4, and 7.5% in the groups D, T and D+T in comparison to control group. Regarding the CV, again groups D, T and D+T showed reductions of 23.5, 13.3, and 12.2%, respectively, in comparison to control group. For this parameter, it was also observed a difference among the treated groups with animals of group D showing a 12.8% lower CV than those from group D+T.

The Vv[glom] was decreased by 43.2, 18.3, and 39.1% in the groups D, T and D+T in comparison to control group. Group T showed higher Vv[glom] than groups D and D+T, with a difference of 43.6 and 34%, respectively. Meanwhile, VWGV of control group was 42.3% higher than that of group D, and 26.1% higher than that of group D+T, but no difference was observed in group T. This latter group showed VWGV 32.3% higher than that of group D.

**Figure 1:**
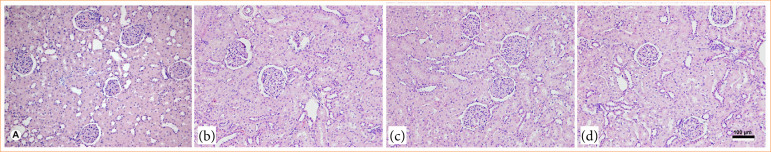
Photomicrographs of (a) renal cortex of control rats, (b) rats treated with dutasteride, (c) tamsulosin, and (d) combined therapy (dutasteride and tamsulosin). Hematoxylin and eosin, 200x.

Source: Elaborated by the authors.

**Figure 2:**
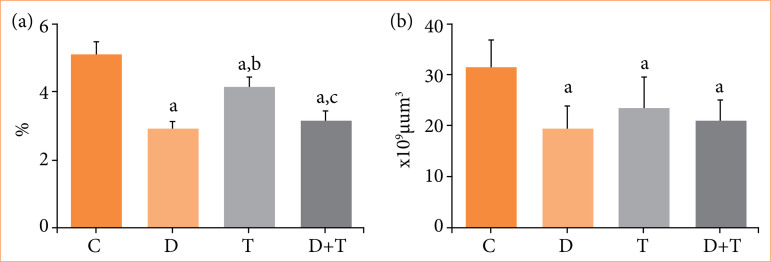
Graphics presenting the results of (a) volumetric density of glomeruli and (b) total number of glomeruli in the kidneys of rats. Data presented as mean ± standard deviation.

Most importantly, the number of glomeruli per kidney was reduced by 37.9% in the group D, by 25.7% in group T, and by 33.07% in group D+T, in comparison to control group. All data is presented in Table 1 and illustrated in Figs. 1 and 2.

**Table 1:** Morphometrical data of kidney from rats that received dutasteride, tamsulosin, or the combination of both drugs**.

	C	D	T	C+T	*p* -value*
Body weight (g)	264.2 ± 5.65	265.4 ± 4.62	264.6 ± 3.95	264.8 ± 3.67	0.9470
Kidney weight (g)	1.30 ± 0.91	1.11 ± 0.05 ^a^	1.16 ± 0.07 ^a^	1.19 ± 0.05 ^a^	< 00001
Kidney volume (mL)	1.23 ± 0.10	1.04 ± 0.07 ^a^	1.10 ± 0.08 ^a^	1.12 ± 0.04 ^a^	< 00001
Cortical-to-medullary ratio (%)	82.14 ± 2.69	72.83 ± 2.38 ^a^	74.40 ± 2.83 ^a^	75.96 ± 1.85 ^a^	< 00001
Cortical volume (mL)	0.98 ± 0.11	0.75 ± 0.06 ^a^	0.82 ± 0.07 ^a^	0.86 ± 0.04 ^a,b^	< 0.0001
Vv[glom] (%)	5.12 ± 0.31	2.91 ± 0.16 ^a^	4.18 ± 0.19 ^a,b^	3.12 ± 0.30 ^a,c^	< 0.0001
VWGV (x10^5^ µm^3^)	16.35 ± 1.84	11.49 ± 1.34 ^a^	15.20 ± 2.73 ^b^	12.97 ± 1.52 ^a^	< 0.0001
Number of glomeruli per kidney (x10^9^ µm^3^)	31.45 ± 5.08	19.52 ± 4.05 ^a^	23.36 ± 5.92 ^a^	21.05 ± 3.79 ^a^	< 0.0001

C: control group; D: group that received dutasteride; T: group that received tamsulosin; C+T: group that received dutasteride and tamsulosin;

* analysis of variance p-value;

a different from C;

b different from D;

c different from T;

** data presented as mean ± standard deviation;

Vv[glom]: glomerular volumetric density; VWGV: volume-weighted glomerular volume.

Source: Elaborated by the authors.

C: control rats; D: rats treated with dutasteride; T: rats treated with tamsulosin; D+T: rats treated with dutasteride and tamsulosin; a: different from C; b: different from D; c: different from T. Source: Elaborated by the authors.

## Discussion

The present study demonstrated the renal damage produced by the administration of commonly prescribed drugs for BPH treatment. This is the first experiment to show that the combined therapy of dutasteride and tamsulosin can reduce the number of glomeruli in rats. These drugs are well known to be associated with adverse effects such as decreased libido, erectile and ejaculatory dysfunction, and morphological alterations in the corpora cavernosum (which can be permanent) have been documented^5,6,8^. However, information regarding side effects in other organs is scarce, especially in tissues not typically recognized as androgen dependent.

Several conditions can reduce the number of glomeruli, including renal ischemia, radiofrequency ablation, hypertension, diabetes, and stress^17–20,22^. As each nephron has one (and only one) glomerulus, the number of glomeruli can be considered to represent the number of nephrons. Thus, these mentioned conditions are thought to increase the risk of chronic kidney disease. Moreover, the relationship between decreased glomeruli and decreased glomerular filtration rate is well known^23^. This reinforces the importance of the data presented in this study. In fact, the quantification of the number of glomeruli is a useful and sensitive method to morphologically evaluate the renal damage after different conditions.

Some interesting results were observed when comparing the studied groups. All groups receiving drugs showed prejudice in kidney weight, kidney volume, cortical-to-medullary ratio, cortical volume, and Vv[glom]. However, some differences among these groups were noted. The group receiving dutasteride had always the worse result. Although this was not statistically significant for some parameters (kidney weight, kidney volume, and cortical-to-medullary ratio), when analyzing the cortical volume, it was possible to observe that animals of group D+T showed less drastic reduction. Further, for the Vv[glom], the tamsulosin treated group had also a discrepant result from other treated groups, being less affected by the drug administered. Finally, animals receiving tamsulosin (group T) were the only ones that had not presented reduction in VWGV. Taken altogether, it seems that, although all experimental treatments induced renal prejudice, tamsulosin (used alone) was the less harmful option.

The mechanisms by how 5-ARIs disturbs the kidney are still poorly known. By inhibiting the conversion of testosterone into DHT, several functions could be affected. In androgen dependent organs, the deprivation of this hormone promotes important modifications, which are well documented mainly in the prostate and penis^2,5,6,24,25^, but other (non-androgenic dependent) organs were scantily studied. However, although the kidney is not an androgen dependent organ, there is some evidence regarding the influence of androgens on renal function and morphology^26^. Future studies that deepen the knowledge into the mechanisms underlying DHT and 5-ARIs on renal tissue are warranted.

The effects of 5-ARIs on renal morphology has been investigated by different methods and research groups. Treatment with finasteride promoted and increased renal inflammatory evidence and disbalances the cell apoptosis and proliferation ratio^9^. This resulted in tubular fibrosis and glomerulosclerosis^9^, which may explain the reduced number of glomeruli observed in the present study. Other research group showed that rats treated with finasteride has reduced glomerular tuft area, glomerular volume, microvessel density, and vascular endothelial growth factor expression^11^. Further, in a previous study of our group, it was observed that treatment with 5-ARIs (both finasteride and dutasteride) reduced the glomerular volume and the number of nephrons in the rats^10^.

Tamsulosin has been largely used for BPH treatment and for managing uroliths, with no kidney-related adverse effects being previously reported in the literature^12,27^. It was surprising that rats treated with tamsulosin showed such morphological damage. Some possible mechanism by how tamsulosin induces renal damage could be related to its effects on renal blood flow^28^, but future studies are warranted to deeply investigate the effects of tamsulosin on kidney.

Although the renal function was not investigated in the present study, some studies showed that the use of tamsulosin is thought to be safe, without dose modification, even in patients with renal disease^29^. However, renal function can remain unaffected after important glomerular losses, and evidence shows that serum levels of urea and/or creatinine can be maintained under normal values, even after a considerable glomerular loss^30,31^. Although the loss of glomeruli is irreversible^10,30^, unaffected glomeruli can increase their filtration rate, thus keeping the renal biomarkers at a normal level^32^. Regarding the effects of 5-ARIs on renal function, some experimental evidence points that this class of drugs can increase the serum urea and creatinine levels^10^. The present study deepened the knowledge on the effects of BPH treatments on renal morphology.

Based on this rodent study, one important translational aspect arised from its results: the treatment with tamsulosin and/or dutasteride should be accompanied for glomerular disease. Furthermore, since the outcomes with tamsulosin—either alone or combined with dutasteride—were less harmful to kidney than those with dutasteride alone, it may be advisable to avoid using dutasteride as monotherapy in patients for whom renal function is an important concern.

The study has some limitations that should be pointed. Although rodents are widely used, the results of animal studies should not be directly transduced to humans. Future clinical studies focusing on the impact of dutasteride and tamsulosin on renal function during BPH treatment are warranted. Furthermore, studies with longer follow-up periods are required to better understand the long-term renal effects of dutasteride and tamsulosin. Finally, the mechanisms underlying the renal damage caused by the investigated drugs are not fully understood yet. Therefore, given this uncertainty and the study’s limitations, further research is warranted.

## Conclusion

The use of dutasteride and tamsulosin, both isolated and in association, leaded to a loss of nephrons. Dutasteride treated animals showed the more severe glomerular loss than those receiving tamsulosin and the combined therapy.

## Data Availability

All data is presented in this manuscript.
